# A Case of Steroid-Responsive Encephalopathy

**DOI:** 10.7759/cureus.17063

**Published:** 2021-08-10

**Authors:** Alona Kondramashin, Asia Filatov, Jonathan T Grossman, Marc Swerdloff

**Affiliations:** 1 Neurology, Florida Atlantic University Charles E. Schmidt College of Medicine, Boca Raton, USA; 2 Neurology, Boca Raton Regional Hospital/Florida Atlantic University Charles E. Schmidt College of Medicine, Boca Raton, USA

**Keywords:** steroid responsive, hashimoto’s encephalopathy, encephalopathy, hashimotos thyroiditis, anti-tpo antibodies

## Abstract

Hashimoto's encephalopathy (HE), also known as steroid-responsive encephalopathy, is associated with autoimmune-mediated thyroiditis. Onset is typically gradual often with evolution over the course of months. Characteristic symptoms include impaired concentration and memory, delusions, hallucinations, personality changes, incoordination, tremor, hemiparesis, seizures, and speech difficulties. Diagnosis is predicated upon discovery of elevated anti-thyroid antibodies, specifically anti-thyroid peroxidase (TPO) or anti-M antibodies. Some patients may also present with subclinical hypothyroidism but many are euthyroid. Of note, neither thyroid function tests or antibody titers correlate with disease severity. Other common laboratory findings include elevations in sedimentation rate, liver enzymes, and cerebrospinal fluid (CSF) protein. Radiological work-up, including cerebral angiography, is often normal. Successful treatment includes administration of steroids and/or intravenous immunoglobulin (IVIg) or plasmapheresis. We describe a case of a 74-year-old male who presented for evaluation of abrupt onset confusion ultimately determined to be a consequence of autoimmune-mediated thyroiditis.

## Introduction

Hashimoto's encephalopathy (HE), or steroid-responsive encephalopathy, is a disorder associated with autoimmune-mediated thyroiditis. It affects both the nervous and endocrine systems [1]. It is characterized by myoclonus, seizures, and confusion with often fluctuating levels of consciousness. The condition may also be associated with paranoid delusions and hallucinations. Unfortunately, the cause remains unknown [2]. Available literature suggests a pathological inflammatory response as a central feature in the evolution of the disease. Still, a clear association between encephalopathy and Hashimoto's thyroiditis (HT) has yet to be established. It is well established that corticosteroid or other immunosuppressive therapies (e.g. IVIg, plasmapheresis) afford significant improvement, or complete resolution, of symptoms. Its course is variable and may be self-limited without treatment, progressive without treatment, resolve with treatment, or follow a relapsing-remitting course in spite of treatment.

## Case presentation

**Figure 1:**
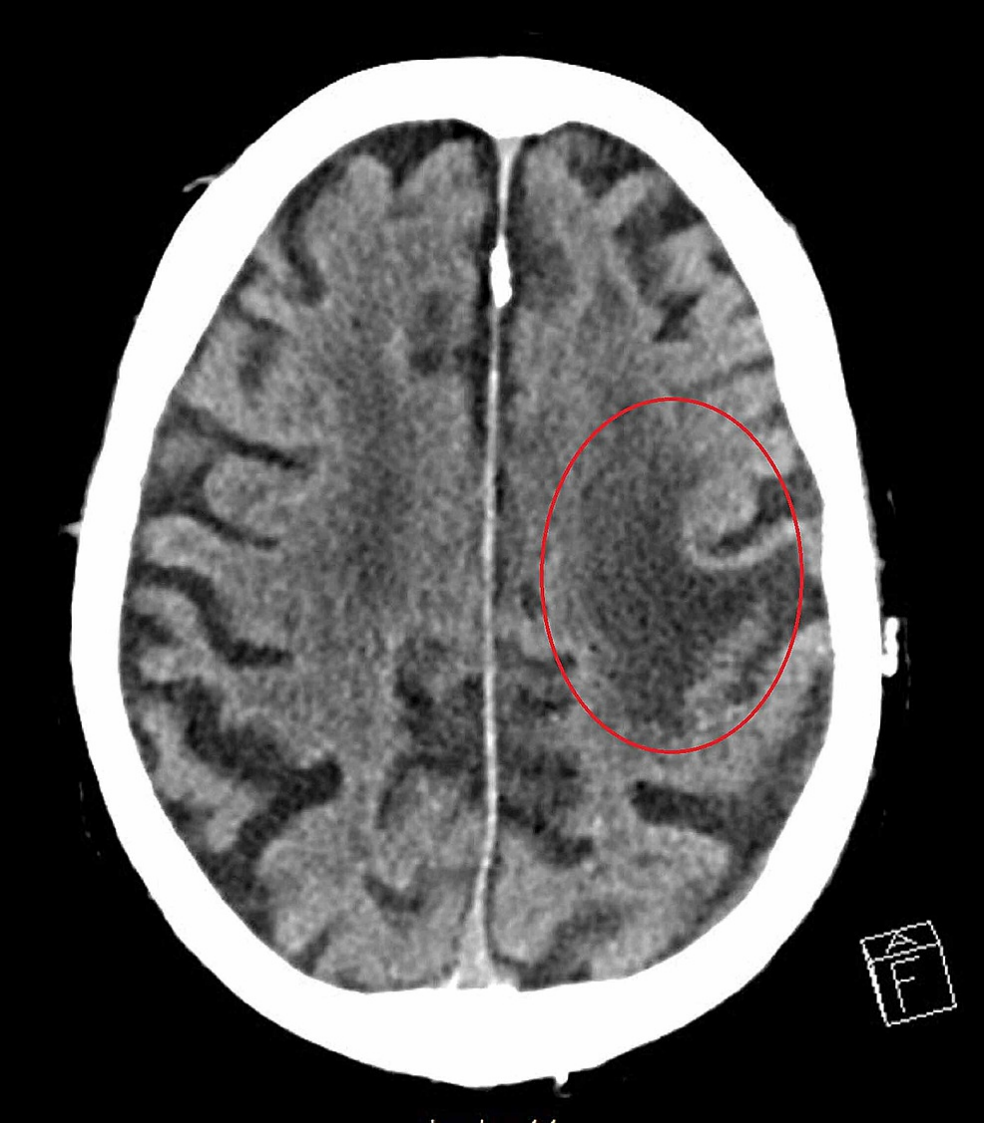
NCCT scan of head showing hypodensity in left frontal lobe representing chronic encephalomalacia. NCCT = non-contrast computed tomography

A 74-year-old male with remote history of left frontal lobe stroke, uncontrolled insulin-dependent diabetes mellitus, coronary artery disease, atrial fibrillation, congestive heart failure, and pseudogout presented for evaluation of waxing and waning confusion that began 24 hours prior to arrival. Prior to evaluation by our Neurology service, he was found to have a urinary tract infection (UTI) and started on Levaquin. A non-contrast computed tomography (NCCT) scan of the head revealed chronic left frontal encephalomalacia (Figure 1).

**Figure 2:**
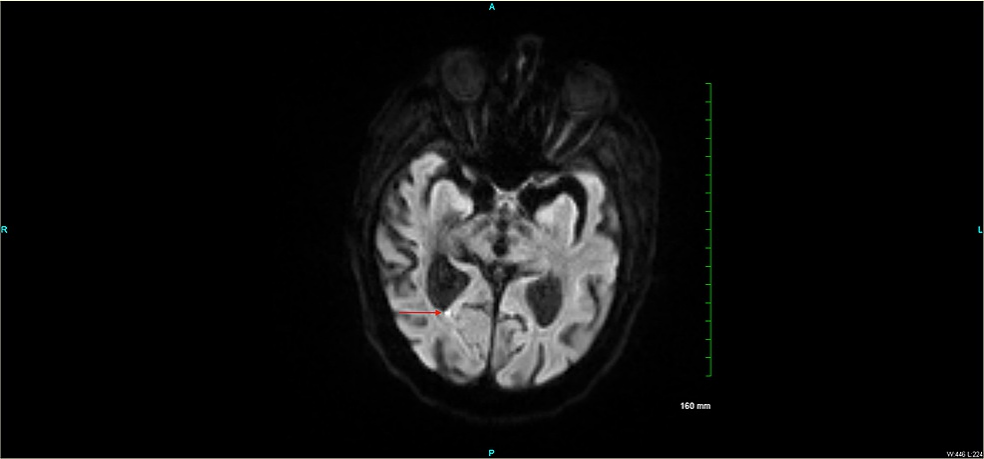
MR axial DWI sequence showing focus of restricted diffusion in the right splenial region of the corpus callosum. MR = magnetic resonance DWI = diffusion weight imaging

Subsequent MRI brain showed an acute infarct in the right splenial region of the corpus callosum on diffusion-weighted imaging (Figure 2).

This infarct was new when compared to an MRI brain that was performed outpatient one week prior to presentation to our emergency department (image unavailable). He had reportedly experienced progressive cognitive decline over the six months preceding presentation.

**Figure 3:**
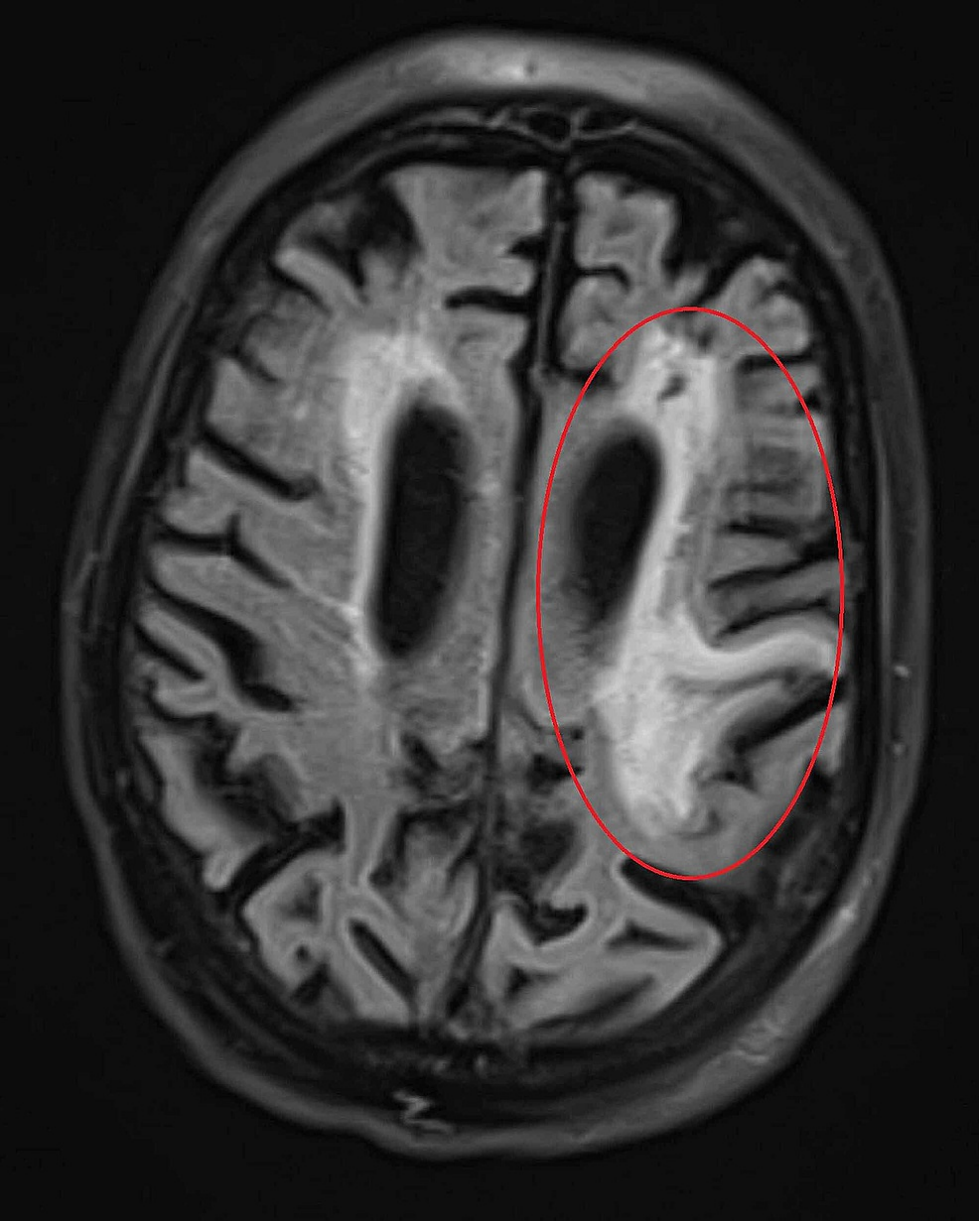
MR axial FLAIR sequence showing increased signal in left frontal lobe representing chronic encephalomalacia. MR = magnetic resonance FLAIR = fluid-attenuated inversion recovery

The finding in Figure 1 was also confirmed via MRI fluid-attenuated inversion recovery (FLAIR) sequence (Figure 3).

**Figure 4:**
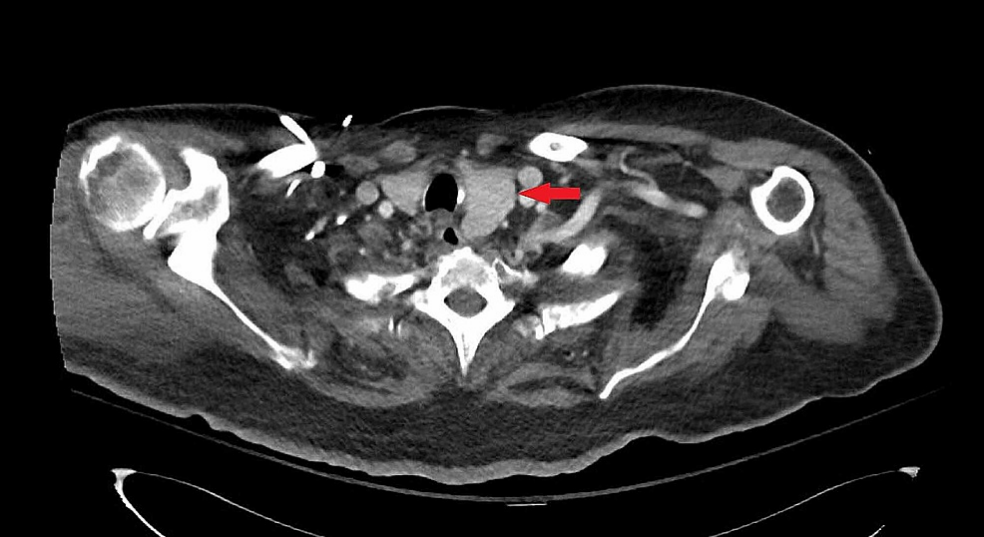
NCCT of the soft tissues of the neck showing prominent left thyroid lobe. NCCT = non-contrast computed tomography

Additional tests were performed to further characterize his persistent stupor. Conventional angiogram was without evidence of vasculitis. Contrast-enhanced CT of the chest showed asymmetric enlargement of the thyroid with leftward sub-sternal extension; there was no sign of malignancy. Contrast-enhanced CT of the abdomen and pelvis was also negative for malignancy. Electroencephalogram was without evidence of epileptiform discharge or seizure activity. A lumbar puncture showed no evidence of pleocytosis but did reveal elevated protein (63mg/dL; normal range 15-45) and IgG index (6.4mg/dL; normal 0.0-6.0). Due to findings on CT chest, NCCT of the soft tissues of the neck was performed which revealed enlarged left thyroid lobe (Figure 4). 

Other notable labs during hospitalization included persistently elevated sedimentation rate with a peak of 127 mm/hr (normal range 0-20), white blood cell count with a peak of 16.3K/µL (normal range 4.5-11.0), C-reactive protein of 10 mg/L (normal range <10), negative blood cultures, total T3 0.3 ng/mL (normal range 0.9-2.44), free T3 1.2 pg/ml (normal range 3.7-4.2), free T4 0.9 ng/dl (normal range 1.3-2.8), thyroid stimulating hormone (TSH) 0.233 mIU/ml (normal range 0.5-1.5), thyroid peroxidase antibody titer 20.1 lU/ml (normal range <2). A paraneoplastic panel and encephalitis screen for herpes simplex virus (HSV) and cytomegalovirus (CMV) were unremarkable.

The elevated thyroid peroxidase antibody titer with concomitant elevation of inflammatory markers suggested a probable diagnosis of HE. 

High-dose steroid infusion was initiated via intravenous solumedrol, 500mg, twice daily for three days. He also received IVIg infusion (0.4mg/kg) once daily for five days. 

Following treatment, his mental status improved rapidly with eventual return to baseline. 

## Discussion

A detailed understanding of the immunopathology underlying HE is paramount in reliable diagnosis. Presently, the mechanism that promotes formation of anti-thyroid antibodies is unknown. Regardless, these antibodies are considered pathologic and may develop as a consequence of cross targeting of foreign antigens invading the body. Still, there is no general consensus in current medical literature [3]. The link between encephalopathy and HT is not yet known since there is no evidence that anti-thyroid antibodies are harmful to neurons [4]. Further, a significant number of encephalopathic patients with antithyroid antibodies are euthyroid. Hence, a more accurate name for this condition has been coined: steroid-responsive encephalopathy associated with autoimmune thyroiditis (SREAT). The type and severity of symptoms vary in those affected by this disease [5]. The common feature in diagnosis of patients with SREAT is the presence of anti-thyroid antibodies, specifically anti-TPO or anti-M antibodies. However, these antibodies have been noted to be highly prevalent in normal subjects. In one study, the prevalence of high anti-TPO antibody concentrations was 11% in euthyroid young adults, 23% in euthyroid women >60 years, and 67% in older women with hypothyroidism [4].

Symptoms

The most common presenting symptom is abrupt confusion with altered level of consciousness but it may be insidious. Some experience lethargy, visual hallucinations, and fluctuating confusion that may eventually lead to dementia [6]. In extreme cases, lethargy can progress to coma [7]. Non-cognitive symptoms of tremor, myoclonus, and seizures may also occur. Still, encephalopathy is the most common symptom. The classic clinical picture is characterized by a decline in cognitive ability with impairment in orientation, level of consciousness, and attention. Associated symptoms include poor appetite, hyperreflexia, seizures, dysarthria, and ataxia. Other attributable, or pathognomonic, symptoms are difficult to identify due to the rare nature of the disorder. Prognostication is difficult owing to the fact that a subset of patients will experience a relapsing-remitting course in spite of treatment [8]. Anxiety, depression, hypersomnia, or difficulty initiating sleep can be the initial presenting symptom [9]. Depression, anxiety, social withdrawal, and emotional lability as sole symptoms have also been reported [10].

Diagnosis

The diagnosis of this disorder is based on specialized clinical tests, history, and identification of characteristic clinical signs and symptoms. Variable presentations make an already difficult diagnosis even more difficult. A detectable level of anti-thyroid antibodies must be present for diagnosis [10]. If high serum levels of these antibodies are found, cerebrospinal fluid should also be evaluated for elevated protein (a nonspecific marker of inflammation that can be observed in patients with underlying endocrine abnormalities). Electroencephalography may detect the presence of subclinical seizure activity or generalized slowing related to encephalopathy. MRI brain is useful to eliminate the possibility of other structural causes of encephalopathy [9].

Treatment

Corticosteroid administration is crucial to establish steroid responsiveness by nonspecifically targeting inflammation including that originating within the central nervous system. An optimal dose of steroids has yet to be established but it is presumed to vary with the severity of the condition. Initially, the general consensus is that high doses of corticosteroids should be used with subsequent taper after the desired result is achieved. High corticosteroid doses over a prolonged period have considerable side effects. Intravenous immune globulin or plasmapheresis may also be used to neutralize circulating autoantibodies [11]. Typically, symptoms are either significantly improved or resolve within a few months of treatment [11].

## Conclusions

A high index of suspicion is necessary for reliable identification and diagnosis of this rare and disabling, but treatable, condition. Routine ordering of anti-thyroid antibodies (i.e. anti-TPO and anti-M) may not be cost effective in the workup of the frequently encountered encephalopathic patient, especially in cases where thyroid function is found to be normal. However, if a patient does not return to baseline in a timely fashion after all potential precipitating factors have been addressed, HE (or SREAT) should be considered.

## References

[1] Liu S, Ma Q, Zheng Y (2019). Febrile Hashimoto's encephalopathy associated with Graves' disease and acute pancytopenia: a case report. Medicine (Baltimore).

[2] Mattozzi S, Sabater L, Escudero D (2020). Hashimoto encephalopathy in the 21st century. Neurology.

[3] Mincer DL, Jialal I (2020). Hashimoto Thyroiditis. https://www.ncbi.nlm.nih.gov/books/NBK459262/.

[4] Fröhlich E, Wahl R (2017). Thyroid autoimmunity: role of anti-thyroid antibodies in thyroid and extra-thyroidal diseases. Front Immunol.

[5] Zaletel K, Gaberšček S (2011). Hashimoto's thyroiditis: from genes to the disease. Curr Genomics.

[6] Anand KS, Garg J, Verma R, Chakraborty A (2014). Hashimoto's encephalitis: unusual cause of reversible dementia. J Family Med Prim Care.

[7] Matsunaga A, Ikawa M, Yoneda M (2019). Hashimoto's encephalopathy. Clin Exp Neuroimmunol.

[8] Yu M, Yang Y, Ma X, Xie Y, Sun N, Meng H (2020). Hashimoto's encephalopathy mimicking viral encephalitis: a case report. Front Neurosci.

[9] Sharma PM, Javali M, Mahale R, Madhusudhan BK, Majeed AA, Srinivasa R (2015). Hashimoto encephalopathy: a study of the clinical profile, radiological and electrophysiological correlation in a tertiary care center in South India. J Neurosci Rural Pract.

[10] Boechat LH, Zollner RL (1999). Reactivity of anti-thyroid antibodies to thyroglobulin tryptic fragments: comparison of autoimmune and non-autoimmune thyroid diseases. Braz J Med Biol Res.

[11] Jiang Y, Tian X, Gu Y, Li F, Wang X (2019). Application of plasma exchange in steroid-responsive encephalopathy. Front Immunol.

